# Snail mediates GDF-8-stimulated human extravillous trophoblast cell invasion by upregulating MMP2 expression

**DOI:** 10.1186/s12964-023-01107-2

**Published:** 2023-05-04

**Authors:** Jiaye Chen, Tinglin Song, Sizhu Yang, Qingxue Meng, Xiaoyu Han, Ze Wu, Jung-Chien Cheng, Lanlan Fang

**Affiliations:** Center for Reproductive Medicine, Henan Key Laboratory of Reproduction and Genetics, The First Affiliated Hospital of Zhengzhou University, 40, Daxue Road, Zhengzhou, Henan, 450052 China

**Keywords:** GDF-8, MMP2, Snail, Trophoblast cells, Invasion

## Abstract

**Background:**

Extravillous trophoblast (EVT) cell invasion is a tightly regulated process that requires for a normal pregnancy. The epithelial-mesenchymal transition (EMT) has been implicated in EVT cell invasion. Growth differentiation factor-8 (GDF-8), a member of the transforming growth factor-beta (TGF-β) superfamily, is expressed in the human placenta and promotes EVT cell invasion by upregulating the expression of matrix metalloproteinase 2 (MMP2). However, the underlying molecular mechanism of GDF-8-induced MMP2 expression remains undetermined. Therefore, the present study aims to examine the role of Snail and Slug, the EMT-related transcriptional regulators, in GDF-8-stimulated MMP2 expression and cell invasion in HTR-8/SVneo human EVT cell line and primary cultures of human EVT cells.

**Methods:**

HTR-8/SVneo and primary cultures of human EVT cells were used to examine the effect of GDF-8 on MMP2 expression and explore the underlying mechanism. For gene silencing and overexpression, the HTR-8/SVneo cell line was used to make the experiments more technically feasible. The cell invasiveness was measured by Matrigel-coated transwell invasion assay.

**Results:**

GDF-8 stimulated MMP2 expression in both HTR-8/SVneo and primary EVT cells. The stimulatory effect of GDF-8 on MMP2 expression was blocked by the inhibitor of TGF-β type-I receptors, SB431542. Treatment with GDF-8 upregulated Snail and Slug expression in both HTR-8/SVneo and primary EVT cells. The stimulatory effects of GDF-8 on Snail and Slug expression were blocked by pretreatment of SB431542 and siRNA-mediated knockdown of SMAD4. Interestingly, using the siRNA knockdown approach, our results showed that Snail but not Slug was required for the GDF-8-induced MMP2 expression and cell invasion in HTR-8/SVneo cells. The reduction of MMP2 expression in the placentas with preeclampsia (PE) was also observed.

**Conclusions:**

These findings discover the physiological function of GDF-8 in the human placenta and provide important insights into the regulation of MMP2 expression in human EVT cells.

Video Abstract

**Supplementary Information:**

The online version contains supplementary material available at 10.1186/s12964-023-01107-2.

## Background

The human placenta is a unique and transient organ that is necessary for the development of the embryo and for maintaining a normal pregnancy. At the tips of the placental villi, cytotrophoblast proliferation forms cell columns. Cells at the distal region of the column lose most of their proliferative ability and differentiate into highly invasive extravillous trophoblast (EVT) cells. During early pregnancy, the invasion of EVT cells into the underlying maternal decidua and vasculature is a critical event for placental development and for ensuring a continuous blood supply to the developing fetus throughout pregnancy [1]. Therefore, the process of EVT invasion has to be tightly regulated. Insufficient invasion of EVTs is a hallmark of abnormal placentation and is associated with miscarriage, preeclampsia (PE), and intrauterine growth restriction. On the other hand, uncontrolled trophoblast cell invasion may result in accrete, choriocarcinoma, and hydatiform moles [2, 3].

Although the placenta is normal tissue, the EVT cells exhibit some common features with cancer cells, including high migratory and invasive properties and the ability for immune escape [4]. The epithelial-mesenchymal transition (EMT) is a crucial event that promotes cancer cell migration and invasion [5]. During EMT, the levels of some critical transcriptional regulators, such as Snail and Slug, are upregulated and involved in regulating the expression of EMT-related genes [6]. It is known that members of the transforming growth factor-β (TGF-β) superfamily can induce EMT in many normal and neoplastic cell types. Several members of the TGF-β superfamily are expressed in the human endometrium and placenta and play important roles in the regulation of implantation and placentation through an autocrine/paracrine manner [7, 8]. We have shown that TGF-β1 inhibits human EVT cell invasion by inducing Snail-mediated down-regulation of the expression of vascular endothelial-cadherin (VE-cadherin) [9]. Notably, EMT has now been considered an important process that regulates EVT cell invasion [10].

Growth differentiation factor-8 (GDF-8) was initially identified and named myostatin because the knockout of its expression led to a dramatic increase in muscle mass in mice [11]. Like other members of GDFs, GDF-8 belongs to the TGF-β superfamily. The expression of GDF-8 is detected in the human placenta [12, 13]. Recently, another group and we have determined the pro-invasive effect of GDF-8 in human EVT cells [14–16]. The matrix metalloproteinases (MMPs)-mediated extracellular matrix (ECM) degradation and tissue remodeling are required for the cell invasion. Aberrant expressions of MMPs are associated with placental diseases [17, 18]. Our recent study reports that GDF-8 stimulates human EVT cell invasion by upregulating the expression of MMP2 [15]. GDF-8 has been shown to stimulate human ovarian cancer cell migration by inducing Snail and Slug-mediated downregulation of E-cadherin, the hallmark of EMT [19]. However, whether Snail and Slug can be regulated by the GDF-8 in human EVT remains unknown. Therefore, the present study was designed to examine the effect of GDF-8 on Snail and Slug expression and their involvement in GDF-8-induced MMP2 expression in human EVT cells.

## Materials and methods

### Antibodies and reagents

The MMP2 (#40994), SMAD4 (#38454), Snail (#3895), and Slug (#9585) antibodies were obtained from Cell Signaling Technology. The α-tubulin (#sc-23948) antibody was obtained from Santa Cruz Biotechnology. The recombinant human GDF-8 and TGF-β1 were obtained from R&D systems. The SB431542 was obtained from Sigma.

### Cell culture and reagents

The HTR-8/SVneo cell line was obtained from American Type Culture Collection through an official distributor in China (Beijing Zhongyuan Limited). HTR-8/SVneo is an SV40 large T antigen immortalized first-trimester short-lived EVT cell line [20]. Cells were cultured in a humidified atmosphere containing 5% CO_2_ and 95% air at 37 °C in Dulbecco’s modified Eagle’s medium/nutrient mixture F-12 Ham medium (DMEM/F-12; Gibco) supplemented with 10% FBS (HyClone), 100 U/mL penicillin and 100 μg/mL streptomycin sulfate (Boster).

### Human primary EVT cell isolation and culture

The study received institutional approval and was carried out in accordance with the guidelines from the Zhengzhou University Research Ethics Board. Human EVT cells were isolated from first-trimester (6–9 weeks of gestation) placental tissue explants as previously described [21, 22]. Briefly, chorionic villi were washed with a cold medium and mechanically minced into 1–2 mm fragments. Fragments of the chorionic villi were allowed to adhere for 2–3 days, after which any non-adherent material was removed. These tissue explants were further cultured for 10–14 days to allow EVT cell outgrowth, during which the culture medium was changed every 2 days. EVT cells were separated from the villous explants by brief trypsin digestion. Cells were plated in a 6-well or 12-well plate (2 × 10^4^ cell/cm^2^) without coating and cultured in a humidified atmosphere containing 5% CO_2_ and 95% air at 37 °C in DMEM/F-12 supplemented with 10% FBS, 100 U/mL penicillin, and 100 μg/mL streptomycin sulfate. Isolated primary EVT cells were characterized by the expressions of cytokeratin-7 and HLA-G as described in our previous study [23]. Primary EVT cells were not passaged. Individual primary cultures were composed of cells from one individual patient. Each experiment was repeated at least three times and each time used cells derived from different patients.

### Reverse transcription quantitative real-time PCR (RT-qPCR)

Total RNA was extracted with the TRIzol reagent (Invitrogen) according to the manufacturer’s instructions. RNA (1 μg) was reverse-transcribed into first-strand cDNA with the iScript Reverse Transcription Kit (Bio-Rad Laboratories). Each 20-μL qPCR reaction contained 1X SYBR Green PCR Master Mix (Applied Biosystems), 60 ng of cDNA, and 250 nM of each specific primer. The following primers were used: MMP2, 5'-TAC ACC AAG AAC TTC CGT CTG T-3' (sense) and 5'-AAT GTC AGG AGA GGC CCC AT-3' (antisense); Snail, 5-CCC CAA TCG GAA GCC TAA CT-3' (sense) and 5'-GCT GGA AGG TAA ACT CTG GAT TAG A-3' (antisense); Slug, 5'-TTC GGA CCC ACA CAT TAC CT-3' (sense) and 5'-GCA GTG AGG GCA AGA AAA AG-3' (antisense); and GAPDH, 5'-GAG TCA ACG GAT TTG GTC GT-3' (sense) and 5'-GAC AAG CTT CCC GTT CTC AG-3' (antisense). qPCR was performed on an Applied Biosystems QuantStudio 12 K Flex system equipped with 96-well optical reaction plates. The specificity of each assay was validated by melting curve analysis and agarose gel electrophoresis of the PCR products. All of the RT-qPCR experiments were run in triplicate, and a mean value was used to determine the mRNA levels. RNase-free water and mRNA without RT were used as negative controls. Relative quantification of the mRNA levels was performed using the comparative Ct method with GAPDH as the reference gene and using the formula 2^–∆∆Ct^.

### Western blot

Cells were lysed in cell lysis buffer (Cell Signaling Technology) supplemented with a protease inhibitor cocktail (Sigma). Equal amounts of protein were separated by SDS polyacrylamide gel electrophoresis and transferred onto PVDF membranes. After 1 h of blocking with 5% nonfat dry milk in Tris-buffered saline (TBS), the membranes were incubated overnight at 4 °C with primary antibodies diluted in 5% nonfat milk/TBS. Following primary antibody incubation, the membranes were incubated with appropriate HRP-conjugated secondary antibodies. Immunoreactive bands were detected using an enhanced chemiluminescent substrate (Bio-Rad Laboratories) and imaged with a ChemiDoc MP Imager (Bio-Rad Laboratories).

### Small interfering RNA (siRNA) transfection and Snail overexpression

To knock down endogenous SMAD4, Snail, or Slug, cells were transfected with 50 nM ON-TARGETplus SMARTpool siRNA targeting a specific gene (Dharmacon) using Lipofectamine RNAiMAX (Invitrogen). The siCONTROL NON-TARGETING pool siRNA (Dharmacon) was used as the transfection control. To overexpress Snail, cells were transfected with 1 µg empty pCMV vector or vector encoding a full-length of human Snail (GeneChem) using Lipofectamine 3000 (Invitrogen).

### Invasion assay

Transwell cell culture inserts (8 µm pore size, 24 wells, BD Biosciences) were coated with 1 mg/mL growth factor-reduced Matrigel (BD Biosciences). HTR-8/SVneo (1 × 10^5^ cells/insert) or primary trophoblast cells (5 × 10^4^ cells/insert) in DMEM/F-12 medium supplemented with 0.1% FBS were incubated for 48 h against a gradient of 10% FBS. Non-invasive cells were removed with a cotton swab from the upper side of the membrane. Cells that penetrated the membrane were fixed with cold methanol, stained with crystal violet (0.5%, Sigma) for 30 min, and subsequently washed thoroughly with tap water. Each experiment was performed with triplicate inserts. In each insert, five microscopic fields were photographed under an optical microscope, and the cell number was counted manually.

### Immunohistochemistry

Paraffin-embedded sections (5 μm) obtained from control and PE patients were deparaffinized and rehydrated. Antigen retrieval was conducted by boiling sections in sodium citrate buffer (pH 6.0) for 8 min. Endogenous peroxidase activity was blocked by incubating sections of 3% hydrogen peroxide solution at room temperature for 10 min. After 1 h of blocking with 3% bovine serum albumin in PBS, sections were incubated with specific primary antibodies overnight at 4 °C. Following primary antibody incubation, the sections were incubated with HRP-conjugated secondary antibody. Sections were developed using the Peroxidase/DAB Dako REAL EnVision Detection System (Dako) and counterstained with hematoxylin. Negative control in the absence of a primary antibody was performed in parallel.

### Statistical analysis

The results are presented as the mean ± SEM of at least three independent experiments. All statistical analyses were analyzed by PRISM software. Multiple comparisons were analyzed using one-way ANOVA followed by Tukey’s multiple comparison test. A significant difference was defined as *p* < 0.05. Values that are statistically different from one another (*p* < 0.05) are indicated by different letters. The values with any common letter are not significantly different.

## Results

### GDF-8 stimulates MMP2 expression through ALK5 in both HTR-8/SVneo and human primary EVT cells

**Fig. 1:**
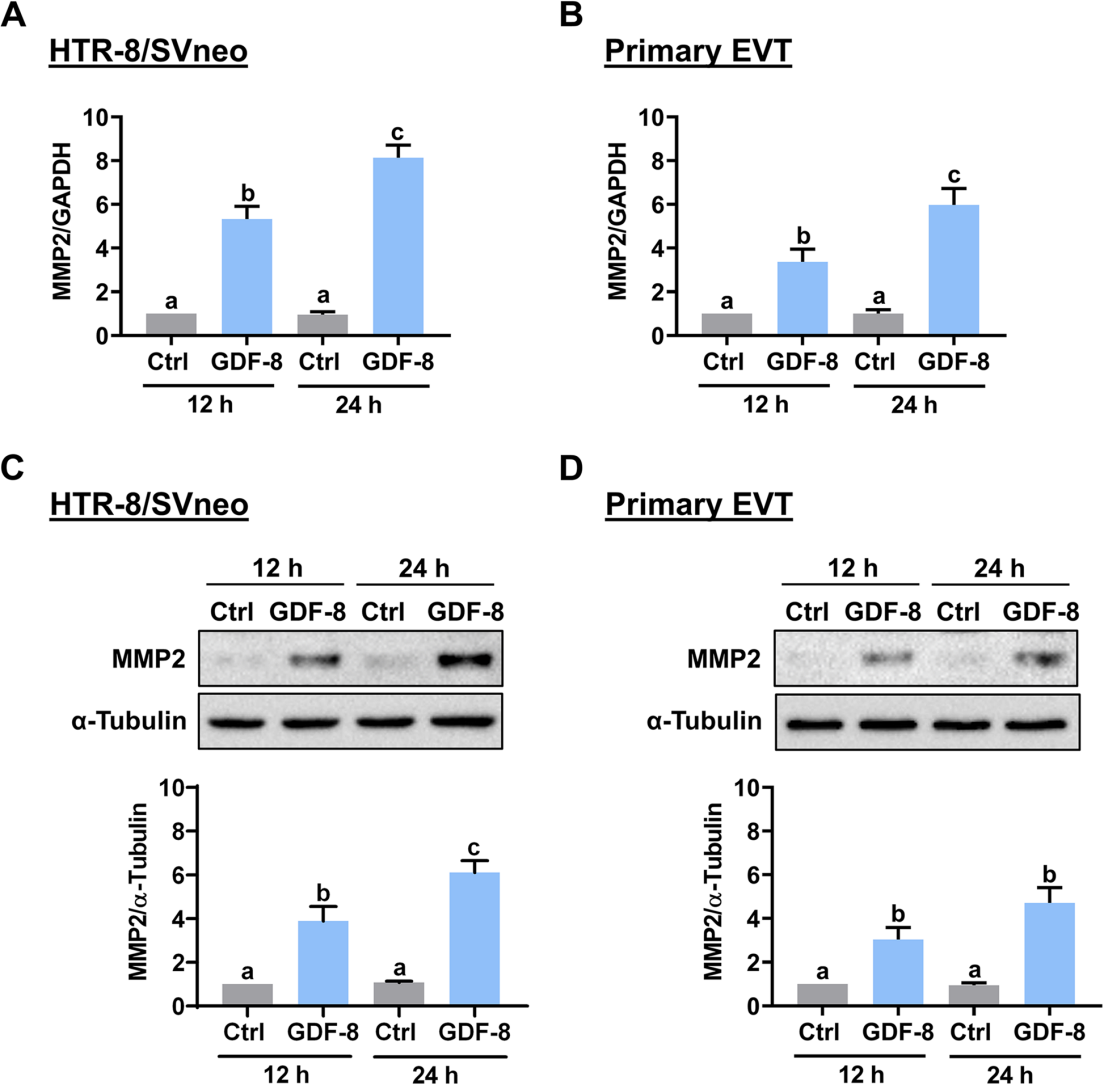
GDF-8 upregulates MMP2 expression in both HTR-8/SVneo and primary EVT cells. **A** and **B**, HTR-8/SVneo (**A**) and primary EVT (**B**) cells were treated with 30 ng/mL GDF-8 for 12 and 24 h. The mRNA levels of MMP2 were examined by RT-qPCR. **C** and **D**, HTR-8/SVneo (**C**) and primary EVT (**D**) cells were treated with 30 ng/mL GDF-8) for 12 and 24 h. The protein levels of MMP2 were examined by western blot. The results are expressed as the mean ± SEM of at least three independent experiments. Multiple comparisons were analyzed using one-way ANOVA followed by Tukey’s multiple comparison test. Values that are statistically different from one another (*p* < 0.05) are indicated by different letters. The values with any common letter are not significantly different

**Fig. 2:**
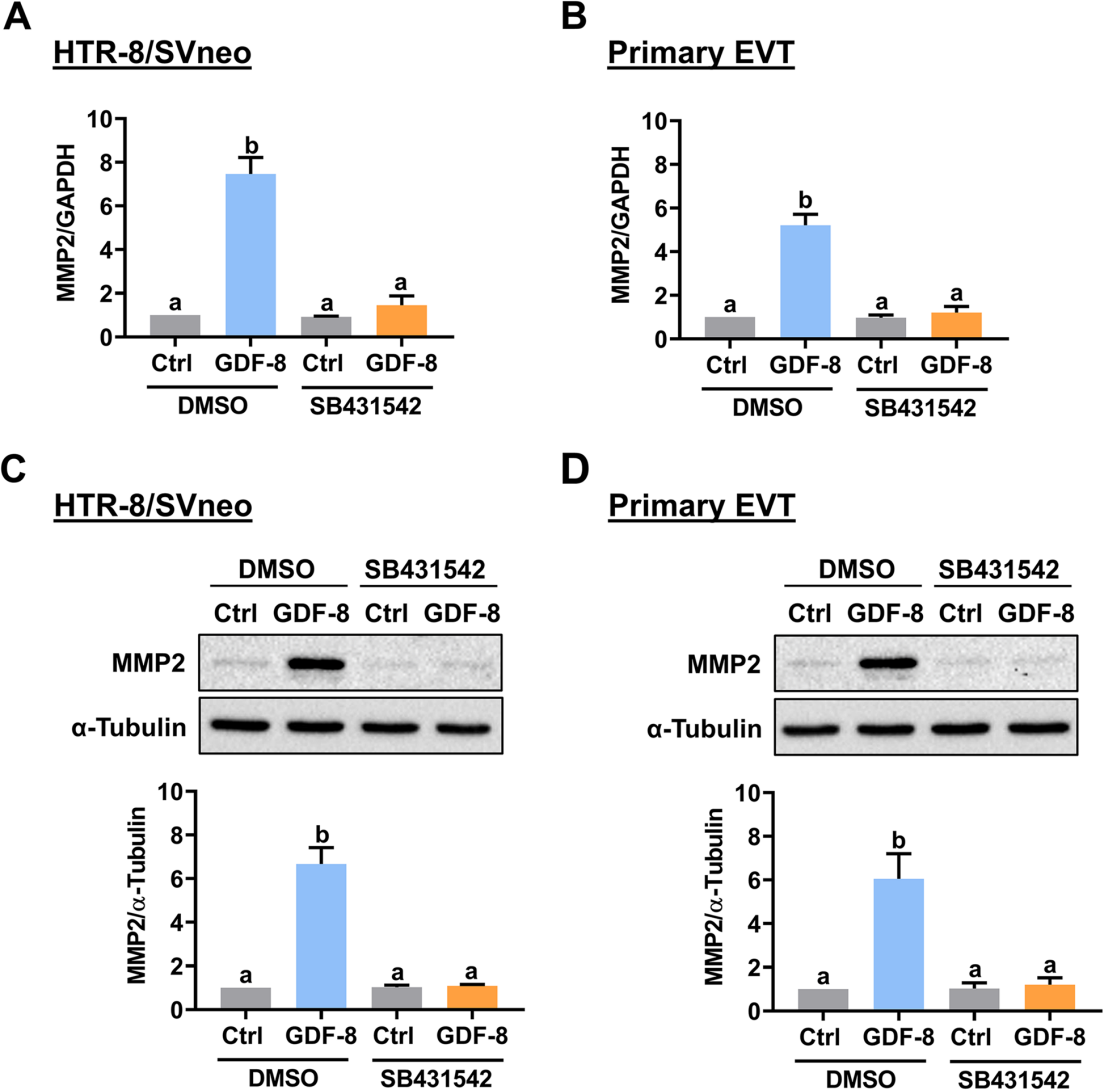
SB431542 blocks the stimulatory effect of GDF-8 on MMP2 expression in both HTR-8/SVneo and primary EVT cells. **A** and **B**, HTR-8/SVneo (**A**) and primary EVT (**B**) cells were pretreated with vehicle control (DMSO) or 10 µM SB431542 for 1 h, and then treated with 30 ng/mL GDF-8 for 24 h. The mRNA levels of MMP2 were examined by RT-qPCR. **C** and **D**, HTR-8/SVneo (**C**) and primary EVT (**D**) cells were pretreated with vehicle control (DMSO) or 10 µM SB431542 for 1 h, and then treated with 30 ng/mL GDF-8 for 24 h. The protein levels of MMP2 were examined by western blot. The results are expressed as the mean ± SEM of at least three independent experiments. Multiple comparisons were analyzed using one-way ANOVA followed by Tukey’s multiple comparison test. Values that are statistically different from one another (*p* < 0.05) are indicated by different letters. The values with any common letter are not significantly different

The HTR-8/SVneo cell line was established by infecting human first-trimester EVT cells with SV40 large T antigen and is a common cell model for studying the biology of EVT cells [20]. To make the experiments more technically feasible, especially for those gene silencing and overexpression, we chose the HTR-8/SVneo cell line as our major experimental model. RT-qPCR results showed that the mRNA levels of MMP2 in HTR-8/SVneo cells were upregulated in response to the treatment of recombinant human GDF-8 for 12 and 24 h (Fig. 1A). In addition, the stimulatory effect of GDF-8 on MMP2 mRNA levels was also observed in the human primary EVT cells (Fig. 1B). Western blot analysis further confirmed the stimulatory effect of GDF-8 on the MMP2 protein levels in both HTR-8/SVneo and human primary EVT cells (Figs. 1C and D). We have shown that the TGF-β type-I receptor, ALK5, mediates the stimulatory effect of GDF-8 on MMP2 expression in HTR-8/SVneo cells [15]. Whether the same is true for human primary EVT cells remains undetermined. As shown in Fig. 2A, consistent with our previous findings, pretreatment of the ALK5 inhibitor, SB431542, did not affect the basal mRNA levels of MMP2 in HTR-8/SVneo cells but blocked the GDF-8-induced upregulation of MMP2 mRNA levels. In human primary EVT cells, inhibition of ALK5 by SB431542 blocked the stimulatory effect of GDF-8 on MMP2 mRNA levels (Fig. 2B). Similar to the RT-qPCR results, pretreatment of SB431542 blocked the GDF-8-induced MMP2 protein levels in both HTR-8/SVneo and human primary EVT cells (Figs. 2C and D).

### GDF-8 upregulates Snail and Slug expression by activating SMAD2/3 signaling pathways

**Fig. 3:**
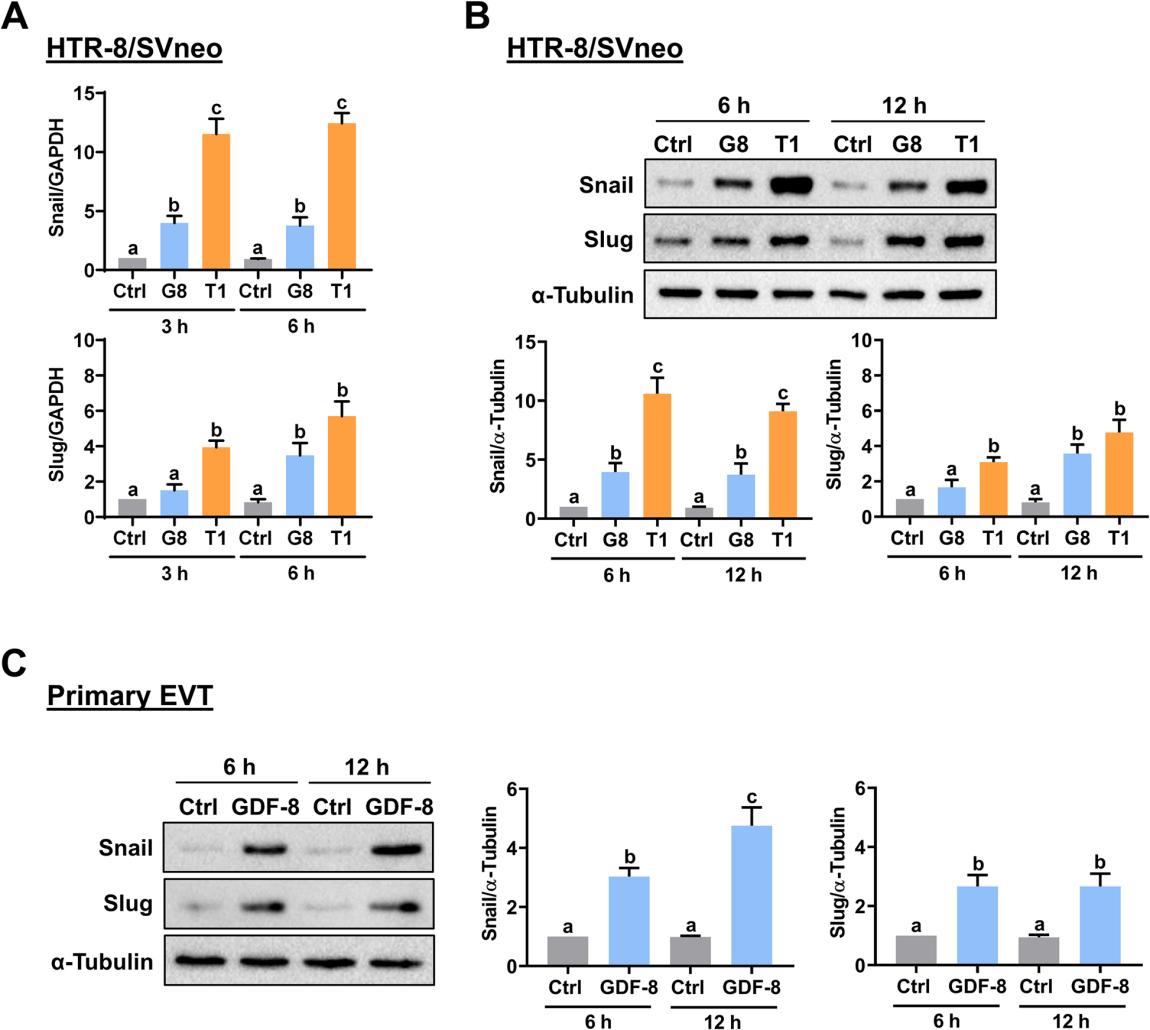
GDF-8 upregulates Snail and Slug expression in both HTR-8/SVneo and primary EVT cells. **A** HTR-8/SVneo cells were treated with 30 ng/mL GDF-8 (G8) or TGF-ꞵ1 (T1) for 3 and 6 h. The mRNA levels of Snail and Slug were examined by RT-qPCR. **B** and **C**, HTR-8/SVneo (**B**) and primary EVT (**C**) cells were treated with 30 ng/mL GDF-8 (G8) or TGF-ꞵ1 (T1) for 6 and 12 h. The protein levels of Snail and Slug were examined by western blot. The results are expressed as the mean ± SEM of at least three independent experiments. Multiple comparisons were analyzed using one-way ANOVA followed by Tukey’s multiple comparison test. Values that are statistically different from one another (*p* < 0.05) are indicated by different letters. The values with any common letter are not significantly different

**Fig. 4:**
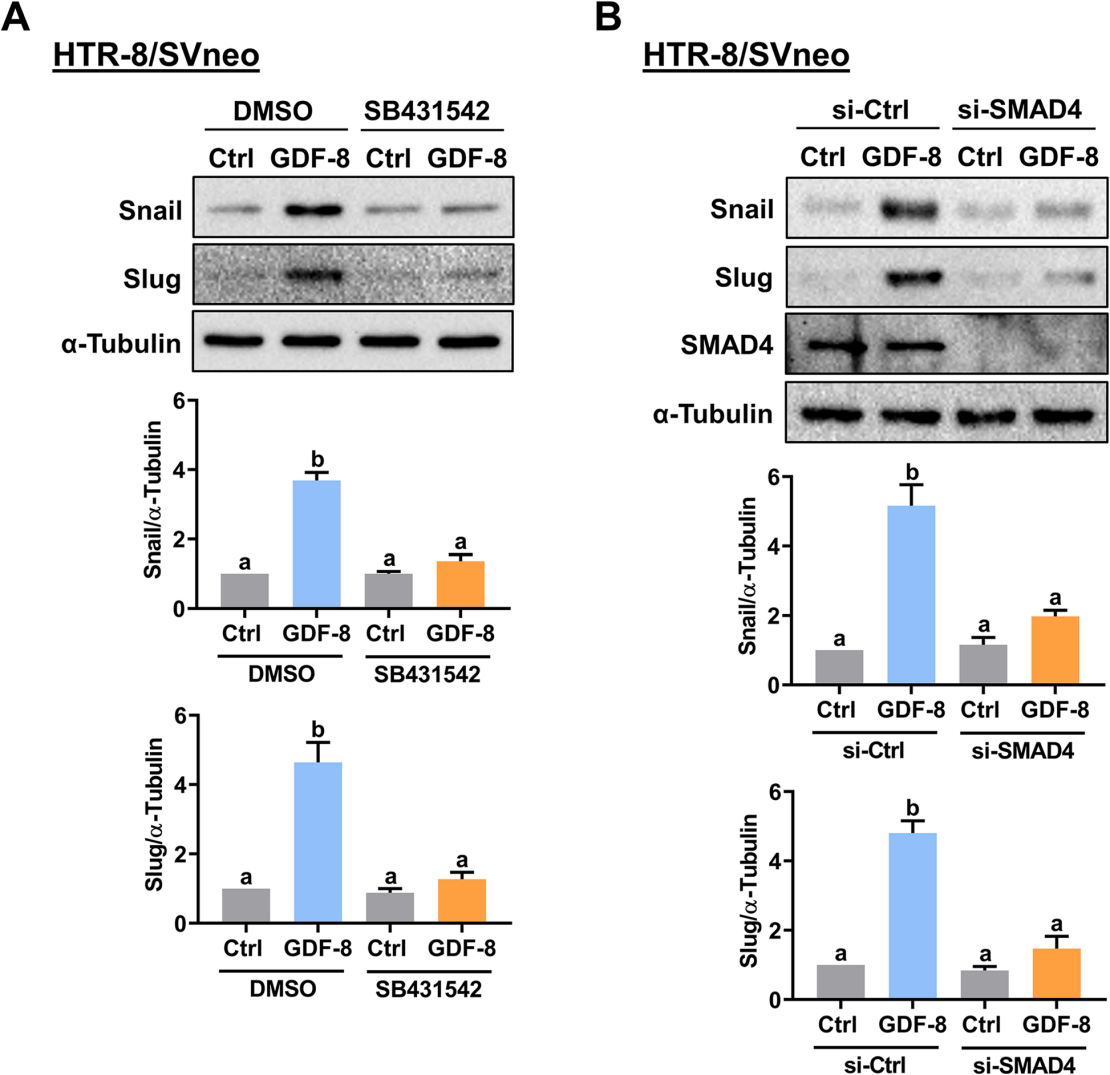
SMAD signaling mediates the stimulatory effects of GDF-8 on Snail and Slug expression. **A** HTR-8/SVneo cells were pretreated with vehicle control (DMSO) or 10 µM SB431542 for 1 h, and then treated with 30 ng/mL GDF-8 for 12 h. The protein levels of Snail and Slug were examined by western blot. **B** HTR-8/SVneo cells were transfected with 50 nM control siRNA (si-Ctrl) or SMAD4 siRNA (si-SMAD4) for 48 h, and then treated with 30 ng/mL GDF-8 for 12 h. The protein levels of Snail, Slug, and SMAD4 were examined by western blot. The results are expressed as the mean ± SEM of at least three independent experiments. Multiple comparisons were analyzed using one-way ANOVA followed by Tukey’s multiple comparison test. Values that are statistically different from one another (*p* < 0.05) are indicated by different letters. The values with any common letter are not significantly different

We have shown that the expressions of Snail and Slug are upregulated in response to the TGF-β1 treatment in HTR-8/SVneo cells [9]. Therefore, we used TGF-β1 as positive controls for the induction of Snail and Slug expression. As shown in Fig. 3A, the treatment of HTR-8/SVneo cells with GDF-8 or TGF-β1 for 3 and 6 h significantly upregulated the mRNA levels of Snail and Slug. The stimulatory effects of TGF-β1 on Snail and Slug mRNA levels were stronger than that of GDF-8. Western blot analysis showed that the protein levels of Snail and Slug in HTR-8/SVneo cells were upregulated by the treatment with GDF-8 or TGF-β1 for 6 and 12 h (Fig. 3B). The inductions of Snail and Slug protein levels by the GDF-8 treatment were also confirmed in the human primary EVT cells (Fig. 3C). In addition, pretreatment of SB431542 blocked the GDF-8-induced upregulations of Snail and Slug protein levels (Fig. 4A). Generally, upon binding to the receptors, GDF-8 activates SMAD2/3 signaling pathways that subsequently mediate the biological functions of GDF-8 [24]. GDF-8 activates both SMAD2 and SMAD3 in HTR-8/SVneo cells [15]. It is well characterized that activated SMAD2 or SMAD3 forms a heterocomplex with the co-SMAD, SMAD4, which is required for regulating the expression of target genes [24]. To examine whether SMAD2/3 signaling pathways are involved in the GDF-8-induced upregulations of Snail and Slug expressions, the siRNA-mediated knockdown approach was applied to block the function of SMAD4. As shown in Fig. 4B, transfection of HTR-8/SVneo cells with SMAD4 siRNA downregulated the endogenous protein levels. In addition, the GDF-8-induced upregulations of Snail and Slug protein levels were attenuated by the knockdown of SMAD4.

### Snail but not Slug mediates the GDF-8-upregulated MMP2 expression

**Fig. 5:**
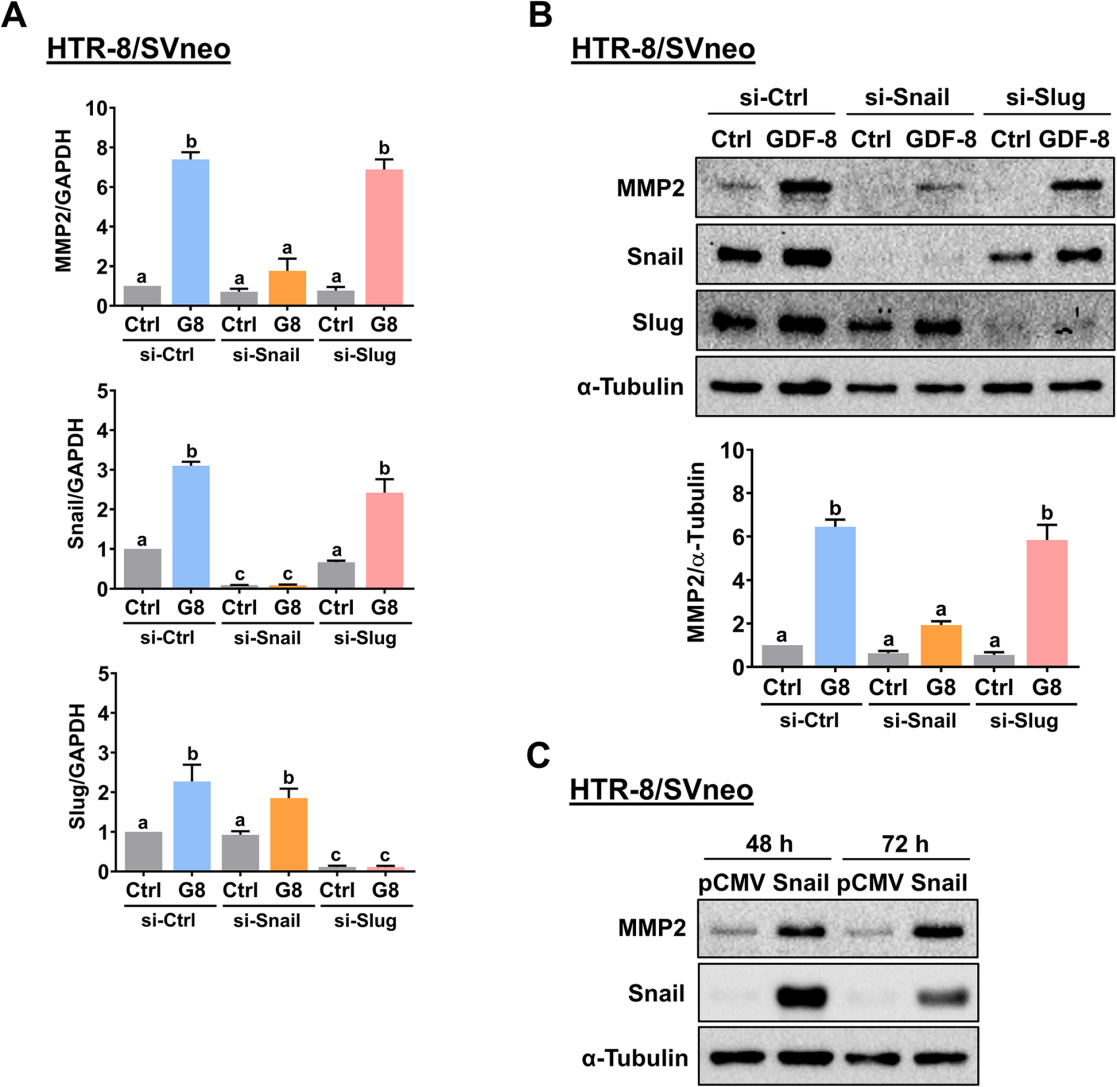
Snail mediates the GDF-8-induced upregulation of MMP2 expression. **A** HTR-8/SVneo were transfected with 50 nM control siRNA (si-Ctrl), Snail siRNA (si-Snail), or Slug siRNA (si-Slug) for 48 h, and then treated with 30 ng/mL GDF-8 (G8) for 6 h. The mRNA levels of MMP2, Snail, and Slug were examined by RT-qPCR. **B** HTR-8/SVneo were transfected with 50 nM control siRNA (si-Ctrl), Snail siRNA (si-Snail), or Slug siRNA (si-Slug) for 48 h, and then treated with 30 ng/mL GDF-8 (G8) for 12 h. The protein levels of MMP2, Snail, and Slug were examined by western blot. **C** HTR-8/SVneo cells were transfected with 1 µg control vector (pCMV) or vector containing Snail (pCMV-Snail) for 48 and 72 h. The protein levels of MMP2 and Snail were examined by western blot. The results are expressed as the mean ± SEM of at least three independent experiments. Multiple comparisons were analyzed using one-way ANOVA followed by Tukey’s multiple comparison test. Values that are statistically different from one another (*p* < 0.05) are indicated by different letters. The values with any common letter are not significantly different

Since GDF-8 induced both Snail and Slug expression, we next used a siRNA-mediated approach to examine the involvement of Snail and Slug in GDF-8-upregulated MMP2 expression in HTR-8/SVneo cells. RT-qPCR results showed that Snail siRNA specifically downregulated the endogenous Snail mRNA levels without affecting the endogenous Slug mRNA levels and vice versa for Slug siRNA in HTR-8/SVneo cells. Interestingly, our results revealed that the GDF-8-induced MMP2 mRNA levels were not affected by the knockdown of Slug but blocked by the knockdown of Snail (Fig. 5A). Western blot analysis showed similar results that Snail but not Slug was required for the GDF-8-upregulated MMP2 protein levels in HTR-8/SVneo cells (Fig. 5B). To further confirm the requirement for Snail in GDF-8-stimulated MMP2 expression, Snail was transiently overexpressed in HTR-8/SVneo cells, and the expression of MMP2 was examined. Western blot analysis showed that cells transfected with Snail for 48 and 72 h resulted in a significant increase in Snail protein levels. Importantly, the protein levels of MMP2 were upregulated in response to the overexpression of Snail (Fig. 5C). Taken together, these results indicated that Snail plays an important regulatory role in mediating the GDF-8-induced MMP2 expression.

### Snail is required for the GDF-8-stimulated human EVT cell invasion

**Fig. 6:**
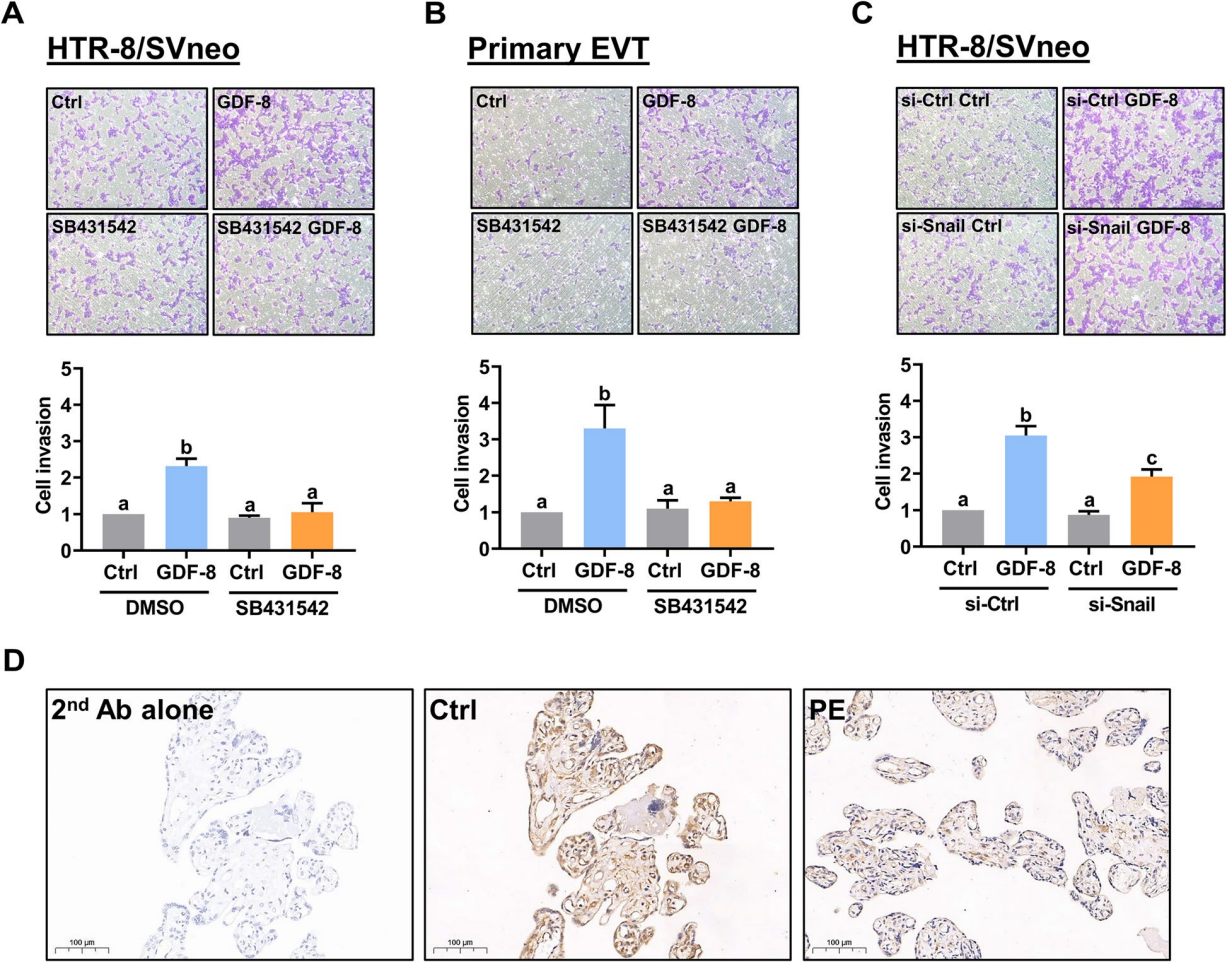
Snail mediates the GDF-8-stimulated cell invasion. **A** and **B**, HTR-8/SVneo (**A**) and primary EVT (**B**) cells were pretreated with vehicle control (DMSO) or 10 µM SB431542 for 1 h, and then treated with 30 ng/mL GDF-8. **C** HTR-8/SVneo were transfected with 50 nM control siRNA (si-Ctrl) or Snail siRNA (si-Snail) for 48 h, and then treated with 30 ng/mL GDF-8. The cell invasiveness was measured by the Matrigel transwell invasion assay. For the invasion assay, the top panel shows representative photos of the invaded cells. The bottom panels show summarized quantitative results. **D** Representative images of immunohistochemical staining for MMP2 in the control and PE placentas. The scale bar represents 100 μm. The results are expressed as the mean ± SEM of at least three independent experiments. Multiple comparisons were analyzed using one-way ANOVA followed by Tukey’s multiple comparison test. Values that are statistically different from one another (*p* < 0.05) are indicated by different letters. The values with any common letter are not significantly different

To examine the effect of GDF-8 on EVT cell invasion, the Matrigel-coated transwell was used. Consistent with the previous study, treatment with GDF-8 stimulated the invasiveness in HTR-8/SVneo cells (Fig. 6A). In human primary EVT cells, similar to the results obtained from HTR-8/SVneo cells, the invasiveness was increased in response to the treatment of GDF-8 (Fig. 6B). The GDF-8-promoted invasiveness was blocked by the inhibition of ALK5 in both HTR-8/SVneo cel primary EVT cells (Fig. 6A and B). In addition, our results showed that siRNA-mediated knockdown of Snail attenuated the GDF-8-stimulated HTR-8/SVneo cell invasion (Fig. 6C). Insufficient EVT cell invasion has been considered a major factor that contributes to the development of PE during pregnancy [25]. Therefore, we next examined the expression of MMP2 in the human placentas. As shown in Fig. 6D, immunohistochemistry analysis showed that the expression of MMP2 protein was detected in both the normal and PE placentas. However, the expression levels of MMP2 protein were decreased in the placentas of PE when compared to normal placentas.

## Discussion

Appropriate regulation of human EVT invasion is necessary for normal placental development. The microenvironment where EVT is located contains a variety of growth factors, hormones, and cytokines that regulate the physiological function of EVT cells in autocrine and paracrine fashions. Immunohistochemistry analysis shows that GDF-8 is expressed in EVT of first- and third-trimester placentas. In addition, expression of GDF-8 is also detected in HTR-8/SVneo cells. Treatment of HTR-8/SVneo and human primary EVT cells with GDF-8 increases cell migration [12]. The cell-adhesion proteins and enzymes for the regulation of ECM remodeling are important molecules that control the invasive processes of the EVT cells. A recent study shows that GDF-8 increases human EVT cell invasion by upregulating N-cadherin via SMAD2/3 signaling pathways [16]. A defining feature of EMT is a reduction in E-cadherin levels and a concomitant induction of N-cadherin [26]. This study supports that the process of EMT is involved in the EVT cell invasion. Our recent study demonstrates that the expression of MMP2 in HTR-8/SVneo cells is upregulated by GDF-8 [15]. In addition to GDF-8, other members of the TGF-β superfamily, such as activin A, GDF-11, and bone morphogenetic protein-2 (BMP-2), have also been reported to promote EVT cell invasion by upregulating the expression of MMP2 [23, 27, 28]. Previous studies have shown that the MMP2 levels are reduced in the placentas of PE patients when compared to those in the placentas of normal women with similar gestation ages [29]. Our results also observed the downregulation of MMP2 expression in PE placentas. Collectively, these results indicate that MMP2 is a critical molecule that mediates EVT cell invasion, and aberrant expression of MMP2 could contribute to poor EVT cell invasion and then lead to the development of PE.

In contrast to the downregulation of MMP2 in PE patients, the serum levels of GDF-8 are significantly higher in PE women (35.2 weeks of gestation) than in the controls (36.8 weeks of gestation). Similarly, the mRNA levels of GDF-8 in the PE placentas (36.2 weeks of gestation) are upregulated when compared to those in the control placentas (38.7 weeks of gestation) [30]. Another study shows that GDF-8 concentrations in the plasma collected at 12–14 weeks of gestation are significantly upregulated in women who later develop PE compared to control. Compared to the gestational age-matched preterm birth placentas (30–32 weeks of gestation), the protein levels of GDF-8 dimer, the active form of GDF-8, are significantly higher in PE placentas [31]. These results suggest that the upregulation of GDF-8 in the PE placentas is likely to be a compensatory effect against insufficient EVT cell invasion. Interestingly, one study reports that the serum levels of GDF-8 at 24–28 weeks of gestation are not significantly varied between PE and control groups [32]. We have shown that serum GDF-8 levels change dynamically during controlled ovarian hyperstimulation in women undergoing in vitro fertilization treatment. It is possible that the contradictory results regarding the alteration of GDF-8 expression in the serum of PE are attributed to the different timings of sampling. Therefore, a further study focusing on the dynamic change of serum GDF-8 during pregnancy will be interesting. The placenta is a heterogeneous and complex organ. There are many factors surrounding sample collection that can influence subsequent analyses obtained from the human placenta [33]. Thus, it is also possible that the different sample collection and preparation methods could lead to those contradictory results. Using single-cell RNA-sequencing, the diversity of the cell type in the human placenta has been revealed [34]. Spatial transcriptomics provides spatially resolved and transcriptome-wide expression information that can be used for study investigating spatial aspects of gene expression [35]. Therefore, to prevent the influence of heterogeneity and different sample collection methods, using single-cell RNA-sequencing together with spatial transcriptomics could help to delineate the specific gene expression levels in a specific cell type of human placenta.

In the present study, our results showed that the expressions of Snail and Slug were upregulated by the treatment of GDF-8 in human EVT cells. These stimulatory effects of GDF-8 on Snail and Slug expressions are consistent with that in human ovarian cancer cells [19]. Snail has been shown to stimulate MMP2 expression in human bone mesenchymal stem cells and hepatocellular cancer cells [36, 37]. Overexpression of Snail increases MMP2 expression by binding to its promoter in squamous cell carcinomas [38]. In ovarian cancer, the expression of Slug is positively correlated with that of MMP2 [39]. Collectively, these studies indicate that Snail and Slug are involved in the regulation of MMP2 expression. However, using a siRNA-mediated knockdown approach, we revealed that the stimulatory effect of GDF-8 on MMP2 expression required Snail but not Slug. Similar to our findings, activin A-induced upregulation of MMP2 expression in human EVT cells is mediated by Snail but not by Slug [27]. Although the requirement of Slug is not examined, a previous study shows that Snail is involved in BMP-2-upregulated MMP2 expression in human EVT cells [28]. Taken together, these findings indicate that Snail plays an important role in mediating the expression of MMP2 in human EVT cells. Interestingly, in human EVT cells, Snail mediates the suppressive effect of TGF-β1 on cell invasion by downregulating the expression of VE-cadherin [9]. AT-rich interactive domain-containing protein 1A reduces cell migration and invasion by downregulating the expression of Snail in the human trophoblast-like JEG-3 cell line [40]. In addition, to date, the alteration of the Snail expression in the placentas of PE remains inconclusive. Downregulation of Snail has been observed in the human PE placentas and in the placentas of the rat PE model [41, 42]. In contrast, other studies show that Snail expression is increased or unaltered in PE placentas when compared to the normal controls [43, 44]. Therefore, the expression of Snail is not always positively associated with EVT cell invasiveness. Depending on the external stimuli, Snail should act as a transcription regulator and cooperate with other induced or activated transcription factors in a context-dependent manner to regulate the expression of different genes, which contributes to either the stimulatory or the inhibitory effect on cell invasiveness.

It is interesting to note that siRNA-mediated knockdown of SMAD4 did not completely block the stimulatory effects of GDF-8 on Snail and Slug expression in HTR-8/SVneo cells. These results suggest that GDF-8-induced Snail and Slug expressions are also mediated by other signaling pathways. In addition to the canonical SMAD signaling pathways, like other members of the TGF-β superfamily, GDF-8 also activates non-canonical signaling pathways such as ERK1/2, JNK, p38 MAPK, and PI3K/AKT signaling pathways in a context-dependent manner [45, 46]. It has been shown that p38 MAPK participates in epidermal growth factor-induced Snail expression in human ovarian cancer cells [47]. In human endometrial cancer cells, Snail can be upregulated by activin B-activated ERK1/2 signaling [48]. We have reported that GDF-8 activates ERK1/2 but not PI3K/AKT in human ovarian granulosa cells [49]. We do not know whether the activation of these non-SMAD signaling pathways can be affected by the GDF-8 in human EVT cells. Future studies investigating SMAD-independent signaling activated by GDF-8 and the functional roles of this signaling in the regulation of Snail expression in human EVT cells will be of interest.

The HTR-8/SVneo cell line is a wildly used cell model for human EVT cells [20]. However, the analysis for the expression of epithelial and mesenchymal markers shows that vimentin, the mesenchymal marker, is detected in a subset of HTR-8/SVneo cells suggesting the presence of the mixed population in this cell line [50]. This is likely due to their transformation/immortalization with SV40 Large T antigen, which is also shown to induce the expression of mesenchymal markers in other primary epithelial cultures [51]. In addition, the expression of vimentin can be detected in interstitial trophoblasts suggesting that the vimentin may not be an exclusive marker of stromal cells within the placenta [52]. Regardless of the purity of the HTR-8/SVneo cell line, using both HTR-8/SVneo and human primary EVT cells, our results showed that Snail expression was upregulated by the GDF-8 and which mediated the GDF-8-stimulated human EVT cell invasion by upregulating MMP2 expression.

## Conclusions

In summary, the present study shows that both Snail and Slug are upregulated in response to the treatment of GDF-8 in both HTR-8/SVneo and human primary EVT cells. Using gain- and loss-of-function approach, our results show that only Snail is required for the GDF-8-induced upregulation of MMP2. In addition, the knockdown of Snail attenuates the stimulatory effect of GDF-8 in HTR-8/SVneo cell invasion. Our study not only discovers the physiological function of GDF-8 in the human placenta but also provides important insights into the regulation of MMP2 expression in human EVT cells.

## Data Availability

The data that support the findings of this study are available from the corresponding author upon reasonable request.

## Abbreviations

BMP: Bone morphogenetic protein
ECM: Extracellular matrix
EMT: Epithelial-mesenchymal transition
EVT: Extravillous trophoblast
GDF: Growth differentiation factor
MMP: Matrix metalloproteinase
PE: Preeclampsia
TBS: Tris-buffered saline
TGF-β: Transforming growth factor-β
VE-cadherin: Vascular endothelial-cadherin
